# Reduction in Vaccine HPV Type Infections in a Young Women Group (18–25 Years) Five Years after HPV Vaccine Introduction in Colombia

**DOI:** 10.1158/1940-6207.CAPR-21-0063

**Published:** 2021-10-05

**Authors:** Alba L. Combita, Viviana Reyes, Devi Puerto, Raúl Murillo, Ricardo Sánchez, Marcela Nuñez, Gustavo A. Hernandez-Suarez, Carolina Wiesner

**Affiliations:** 1 Grupo de Investigación en Biología del Cáncer, Instituto Nacional de Cancerología (INC), Bogotá, Colombia.; 2 Departamento de Microbiología, Facultad de Medicina, Universidad Nacional de Colombia, Bogotá, Colombia.; 3 Grupo de Investigación en Salud Pública y Epidemiología, INC, Bogotá, Colombia.; 4 Centro Javeriano de Oncología, Hospital Universitario San Ignacio, Bogotá, Colombia.; 5 Grupo de Investigaciones Clínicas, INC, Bogotá, Colombia.; 6 GASPI. Grupo Apoyo y Seguimiento para la Investigación, INC, Bogotá, Colombia.

## Abstract

In Colombia, the human papillomavirus (HPV) vaccine was launched in 2012 in the context of a school-based national vaccination program targeting girls ages 9 to 14 and offering catch-up vaccination for girls ages 14 to 17. In this study, we evaluated the program's impact on type-specific HPV infection by comparing HPV cervical prevalence among vaccinated and nonvaccinated women. This is a comparative cross-sectional study conducted 5 years after the quadrivalent HPV vaccination implementation in a sentinel Colombian City. This study included young women (18–25 years old) who had been vaccinated in the catch-up group and were attending universities and technical institutions, and women who attended primary health care facilities for Pap smear screening. The HPV prevalence of 1,287 unvaccinated women was compared with the prevalence of 1,986 vaccinated women. The prevalence of HPV16/18 infections was significantly lower in vaccinated compared with unvaccinated women (6.5% vs. 15.4%; *P* < 0.001), whereas for HPV6/11 infections, a decrease of 63.7% in vaccinated women (1.02% vs. 2.81%) was observed. The adjusted effectiveness to HPV16/18 was 61.4%; 95% CI, 54.3%–67.6%. However, the effectiveness against HPV16/18 was significantly higher among women vaccinated before their sexual debut 91.5%; 95% CI, 86.8–94.5, compared with effectiveness for vaccination after their sexual debut, 36.2%; 95% CI, 23.6–46.7. Five years after the introduction of HPV vaccines in Colombia, high effectiveness of HPV to prevent HPV16/18 infections is observed in the catch-up cohorts including virgin and sexually active women.

**Prevention Relevance:**

Monitoring HPV vaccines post-licensure plays an important role in assessing the progress of immunization programs, demonstrating the impact of vaccines on the population, and providing data for policy needs. In Colombia, HPV vaccines showed effectiveness when administered before start of sexual activity, and two doses are sufficient to achieve good protection.

## Introduction

National human papillomavirus (HPV) vaccination programs have been implemented in many countries with coverage ranging from 20% to 70% (1). Even though the modes of administration and organization may vary among countries, the results of these programs have confirmed that HPV vaccines are effective in preventing HPV infection and HPV-related cervical disease associated with HPV vaccine types (2). In Colombia, the quadrivalent HPV vaccine was included in August 2012, through the Colombian National Immunization Program (CNIP) Schedule. The vaccine was offered to girls ages 9 to 12 years old through a school-based delivery strategy with a three-dose immunization schedule (0–2–6 months).

From 2013 onward, the target age was expanded to 9 to 17 years old (catch-up group) and the original immunization schedule was modified to an alternative one (0–6–60 months; ref. 3). But, since 2018, following WHO recommendations, it was modified to two doses (0/6 months).

From August 2012 to April 2014, the coverage with two doses of HPV vaccines was estimated at over 90% (4, 5). However, in May 2014, there was an increasing number of suspected adverse effects in a small village in the north of the country, where a group of predominantly 13- to 15-year-old girls experienced a variety of clinical symptoms consistent with an episode of Mass Psychogenic Illness (MPI). Subsequently, as an effect of the news and social media, a marked decline in HPV vaccination uptake occurred in the whole country (6), and despite the fact the outbreak analysis found no association between HPV vaccination and the reported adverse events, national vaccination coverage remains below 20% (4, 6, 7). Thus, restoring public confidence in HPV vaccines is a major goal for cervical cancer control in Colombia, where data on safety and effectiveness are highly desirable.

With the introduction of HPV vaccination, a variation in the dynamics of HPV transmission is expected. Therefore, HPV monitoring programs to assess changes in HPV prevalence and patterns of HPV-associated disease are highly required. Population-based cohort studies represent the most accurate design to assess the impact of HPV vaccination (8). However, these designs have limited feasibility in low- and middle-income countries due to their costs and requirements for information systems in the long-run. Thus, cross-sectional surveys in sentinel populations might be a suitable alternative (9), particularly if HPV detection is not part of routine care or screening.

In a previous cross-sectional survey, a high prevalence of HPV infections among young women under 25 years with normal cervix in a sentinel population in Colombia was observed, making this age group an appropriate target population to estimate the short-term impact of HPV prophylactic vaccines (10). Accordingly, to establish the impact of the Colombian HPV vaccination program, we conducted a new cross-sectional analysis to determine the prevalence of vaccine and nonvaccine HPV types among 18- to 25-year-old sexually active women as related to their history of HPV vaccination.

## Materials and Methods

### Study population

This is a comparative cross-sectional study conducted 5 years after the introduction of the HPV quadrivalent vaccine in Manizales, a sentinel city in Colombia. The city was selected because its well-structured population-based cancer registry allows long-term follow-up to evaluate the effectiveness of HPV vaccination. The study included 18- to 25-year-old women corresponding to the catch-up group of the National Immunization Program. Women were voluntarily and consecutively recruited by an open invitation in colleges and primary health care facilities for cervical cancer screening. The invitation was carried out by advertising on local radio, pamphlets, and flyers, as well as by in-person invitation to groups in college classrooms.

Vaccinated women were eligible if they have had at least one dose of the HPV vaccine and the elapsed time between the last dose and the cervical smear collection was longer than a year. Exclusion criteria for vaccinated and not vaccinated included pregnancy, history of hysterectomy, mental impairment or no sexual onset at the time of the study. All women signed informed consent. Sociodemographic characteristics, data on sexual and reproductive health, and status of HPV vaccination were collected in a self-administered questionnaire. HPV vaccination status was verified with the vaccination card at sample collection or via the web in the nominal registry of the National Immunization Program (PAIWEB by the MSPS). Data were captured online in Redcap.

Cervical smears from unvaccinated women were collected between May 2014 and February 2015 as part of a baseline group for further comparisons (10), and smears from those vaccinated for the present survey were collected between January 2016 and December 2018.

### Sample collection and pap smear testing

Thereafter, each woman underwent a gynecologic examination, and two cervical samples (one each for Pap and HPV testing) were obtained as it was previously published (10). The cytological slides were then referred to a certified laboratory for Pap smear testing. The slides were stained with Papanicolaou stain and were later evaluated by a pathologist-supervised cytotechnologist, and classified according to the 2014 Bethesda System. As a quality control procedure, an expert pathologist once again evaluated the positive cervical smears and 10% of negative cervical smears.

### DNA extraction from cervical samples and HPV genotyping

DNA extraction was performed using the QIA-cube with the AmpliLute Liquid Media Extraction kit, following the manufacturer's instructions (Roche Diagnostics), and subsequently, the HPV detection and genotyping were assessed with the Linear Array HPV Genotyping Test (Roche Diagnostics), which detects 13 high- and 23 low-risk HPV types, as it was previously published (10).

### Statistical analysis

The HPV type–specific prevalence was estimated as the proportion of participants who tested positive for a given HPV type. Prevalence for unvaccinated and vaccinated women were estimated as follows: any HPV type, any high-risk HPV type (HR-HPV); any low-risk HPV-type (LR-HPV); nonvaccine HR-HPV types, HPV6–11–16–18, HPV16–18, HPV6–11, and HPV31 and 45, as there is some evidence of cross-protection on them.

Given the differences between unvaccinated and vaccinated women, a propensity score matching based on inverse probability of treatment weighting was performed to balance differences in covariates between the two groups (11–13). The covariates included were age, socioeconomic stratum, residence area, marital status, smoking, age of sexual debut, number of sexual partners, occasional sexual partners, contraceptive method, and history of sexually transmitted diseases.

Comparisons in demographic characteristics, HPV prevalence rates, and cytological results between the two groups were performed using the chi-square test for categorical data. The propensity score was used to balance differences in baseline covariates between unvaccinated and vaccinated women in total and stratified by vaccination status. Statistical analysis was performed using R-project v4.0.0 (free license, R Core Team, 2020). The covariate balance for the propensity score matching was done with the cobalt package in R (14).

Cytological results were stratified according to the 2014 Bethesda System (TBS). Comparisons of HPV types prevalence according to Pap smear results between the groups were performed. An ordinal logistic regression model was used to adjust prevalence estimates after propensity score matching (15).

Vaccine effectiveness was estimated as 100 × [1 − odds ratio] with the corresponding 95% confidence intervals (95% CI). Effectiveness was also estimated according to sexual debut and the number of vaccine doses. The level of significance was *P* < 0.05.

## Results

### Description of study population

A total of 3,465 women ages 18 to 25 years were invited, and 3,273 were included in the study: 1,426 of them were unvaccinated (41.15%) and 2,039 vaccinated (58.84%). Of the 1,426 nonvaccinated women, 139 were excluded due to samples not suitable for HPV typing (59 women), B-globin negative (38 women), and no cytology report (42 women). Among vaccinated women, 53 were excluded because they did not meet the inclusion criteria. The final population for analysis corresponds to 1,287 (39.3%) unvaccinated and 1986 (60.7%) vaccinated women.

Among vaccinated women, 33.4% (664/1,986) received at least one dose of vaccine, 63.9% (1,269/1,986) received two doses, and only 2.7% (53/1,986) went on to complete the full three-dose vaccination schedule. 53.5% (1,062/1,986) of them had received their first dose before sexual debut.

The characteristics of the study population are described in Table 1. Vaccinated women were on average younger than unvaccinated women (mean age, 19 and 22 years, respectively) and were more often single (90.7% vs. 70.9%), living in an urban area (98.1% vs. 92.1%), with less smoking habit (92.4% vs. 77.8%) than unvaccinated women. Between 68.8% and 73.2% of participants reported sexual initiation between ages 14 and 17 years. However, vaccinated women reported having more than four lifetime male partners (21.7%), and more than one occasional sex partner (44.8%). To balance differences in covariates between two groups, we used propensity scoring analysis to eliminate differences between unvaccinated and vaccinated groups. The *P* values after propensity score adjustment were all >0.05, and the standardized differences for most of the variables were <20%, which indicates that variables were successfully balanced (Table 1; Supplementary Fig. S1).

**Table 1.** Characteristic of the study population. Comparison between vaccinated and unvaccinated women ages 18 to 25 years by sociodemographic and sexual behavior characteristics.

	Unvaccinated		Vaccinated			
	*n* = 1,287		*n* = 1,986				
Characteristic	*N* (%)	Median	*N* %	Median	*P*	*P*_Adj_ ^a^
**Age**		22 (20–23)		19 (18–21)	0.00	0.909
**Marital status**						
Single	913 (70.9)		1,801 (90.7)		0.00	0.731
Married	43 (3.3)		29 (1.5)			
Divorced/separated	12 (0.9)		1 (0.1)			
Free union	318 (24.7)		154 (7.8)			
Widow	1 (0.1)		1 (0.1)			
**Residency area**						
Urban	1,157 (92.1)		1,948 (98.1)		0.00	0.403
Rural	99 (7.9)		38 (1.9)			
**Socioeconomic stratum**						
Low	783 (62.3)		914 (46.0)		0.00	0.750
Middle	371 (29.5)		724 (36.4)			
High	102 (8.1)		348 (17.5)			
**Smoking habit**						
Yes	286 (22.2)		150 (7.6)		0.00	0.563
No	1,000 (77.8)		1,836 (92.4)			
**Age sexual debut**						
<14	110 (8.5)		133 (6.7)		0.09	0.816
14–17	885 (68.8)		1,454 (73.2)			
18+	292 (22.7)		399 (20.1)			
**Sex partners**						
1	280 (22.0)		480 (24.2)		0.00	0.687
2–4	785 (61.6)		1,076 (54.2)			
>4	210 (16.5)		430 (21.7)			
**Occasional sex partners**						
None	883 (70.8)		1,098 (55.3)		0.00	0.777
1–2	213 (17.1)		665 (33.5)			
3–5	116 (9.3)		160 (8.1)			
>5	35 (2.8)		63 (3.2)			
**Contraceptive method**						
Intrauterine device	68 (6.2)		328 (17.9)		0.00	0.875
Hormonal	1,083 (81.4)		1,581 (77.9)			
Tubal ligation	89 (8.1)		50 (2.7)			
No use	47 (4.3)		27 (1.5)			
**Diagnostic of STDs** ^b^						
Yes	123 (9.5)		106 (5.4)		0.00	0.351
No	1,164 (90.5)		1,880 (94.6)			

^a^Adjusted by using a propensity score analysis.

^b^STD, sexually transmitted diseases.

### HPV prevalence and type distribution

**Figure 1.**
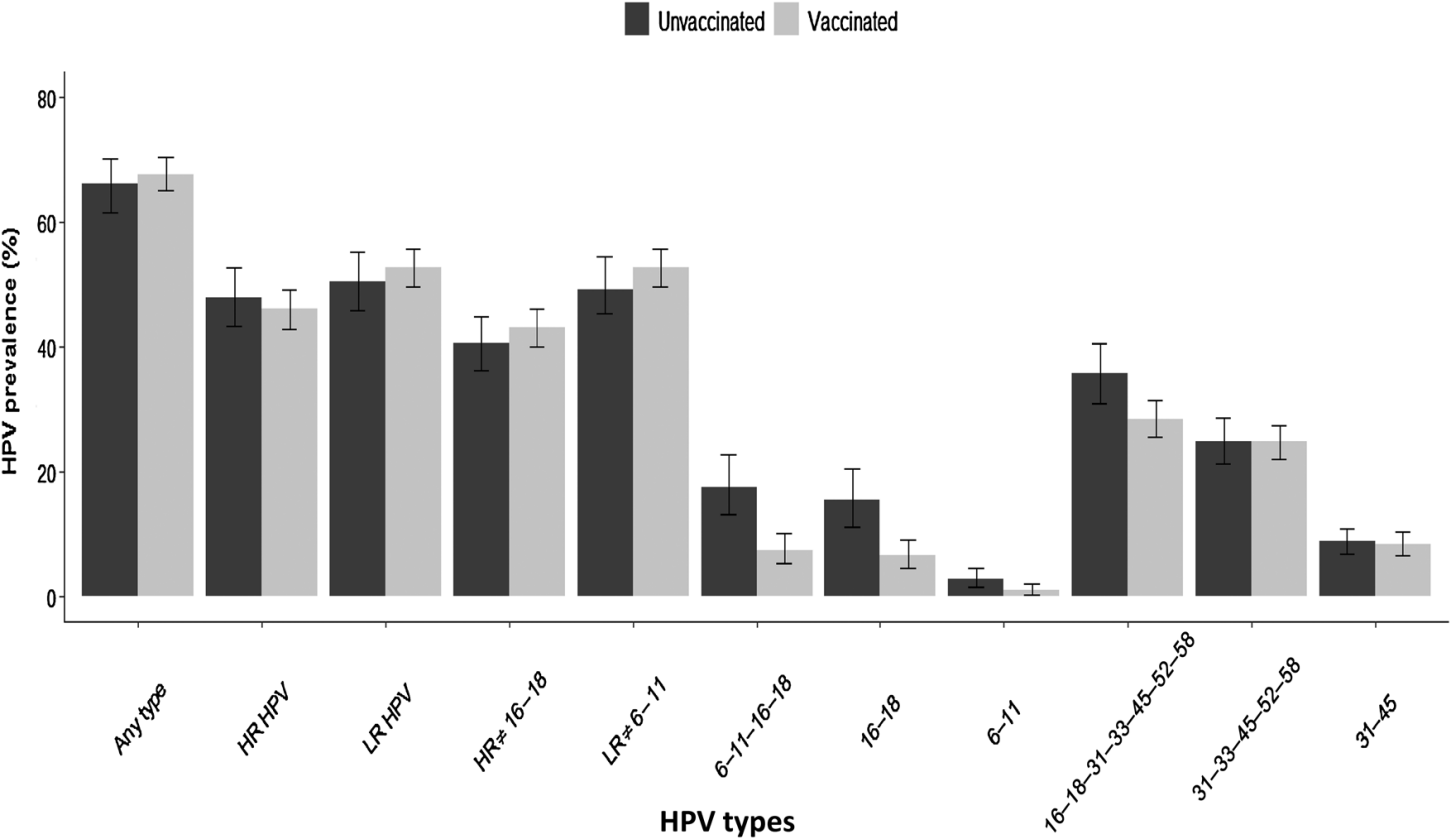
Prevalence of HPV types in the study population. Comparison between unvaccinated and vaccinates women ages 18 to 25 years. Adjusted by using a propensity score analysis.

**Figure 2.**
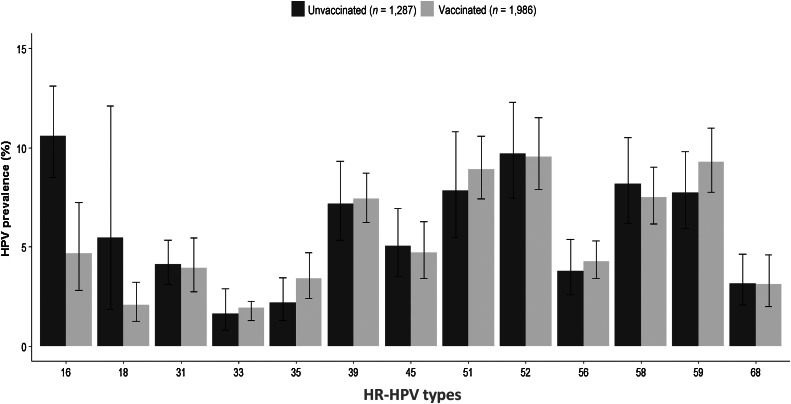
Prevalence of HR-HPV types in the study population. Comparison between unvaccinated and vaccinates women ages 18 to 25 years. Adjusted by using a propensity score analysis.

After balancing by using the propensity score, no significant differences in prevalence of any HPV were observed between unvaccinated and vaccinated women (66.0% vs. 68.0%, respectively, *P* = 0.31). Likewise, no significant differences in prevalence of non-HPV16–18 between unvaccinated and vaccinated women (40.4% vs. 43.0% respectively, *P* = 0.17), and non 6–11 HPV types (49.0% vs. 52.6%, *P* = 0.14) were observed (Fig. 1). The most frequent HR-HPV type in unvaccinated women was HPV16, followed by types 52, 58, 51, and 59. On the other hand, HPV52 was the most frequent type in vaccinated women, followed by types HPV59, 51, and 58 (Fig. 2; Supplementary Table S1). Regarding LR-HPV, a light increase in the prevalence of some types was observed in the vaccinated women group, which was nonsignificant (50.4% vs. 52.6%, *P* = 0.24). However, a significant increase in HPV62 (7.12%–10.12%, *P* < 0.001), HPV84 (7.12%–9.41%, *P* < 0.001), HPV42 (5.19%–8.61%, *P* < 0.001), and HPV67 (2.04%–3.78, *P* < 0.001) was observed in vaccinated women (Supplementary Fig. S2 or Supplementary Table S2).

Regarding HR-HPV vaccine types, the proportion infected with HPV16 changed significantly in vaccinated women, from 10.6% to 4.66% (56.6% decline; *P* < 0.001), while for HPV18 it dropped from 5.45% to 2.09% (61.6% decline; Fig. 2; Supplementary Table S1). Similarly, in relation to LR-HPV vaccine types, there was a significant decrease in the prevalence of HPV 11 in vaccinated women, from 0.97% to 0.0% (*P* < 0.001). As for HPV6, a slight decrease was observed (from 1.86% to 1.02%), though it was not significant, *P* = 0.06; Supplementary Fig. S2; Supplementary Table S2). Overall, a reduction of 63.5% for all vaccine types, from 17.6% to 7.4%, was observed (Fig. 1).

Regarding related HR-HPV types, no significant differences in prevalence for HPV31 and HPV45 in unvaccinated versus vaccinated women were observed [4.13% vs. 3.94% (*P* = 0.83) and 5.03% vs. 4.71% (*P* = 0.66), respectively; Fig. 2; Supplementary Table S1]. Likewise, no significant differences were detected for HR-HPV nonavalent vaccine types, including HPV31, 33, 45, 52, and 58 (24.9% vs. 24.7%, *P* = 0.97; Fig. 1).

**Figure 3.**
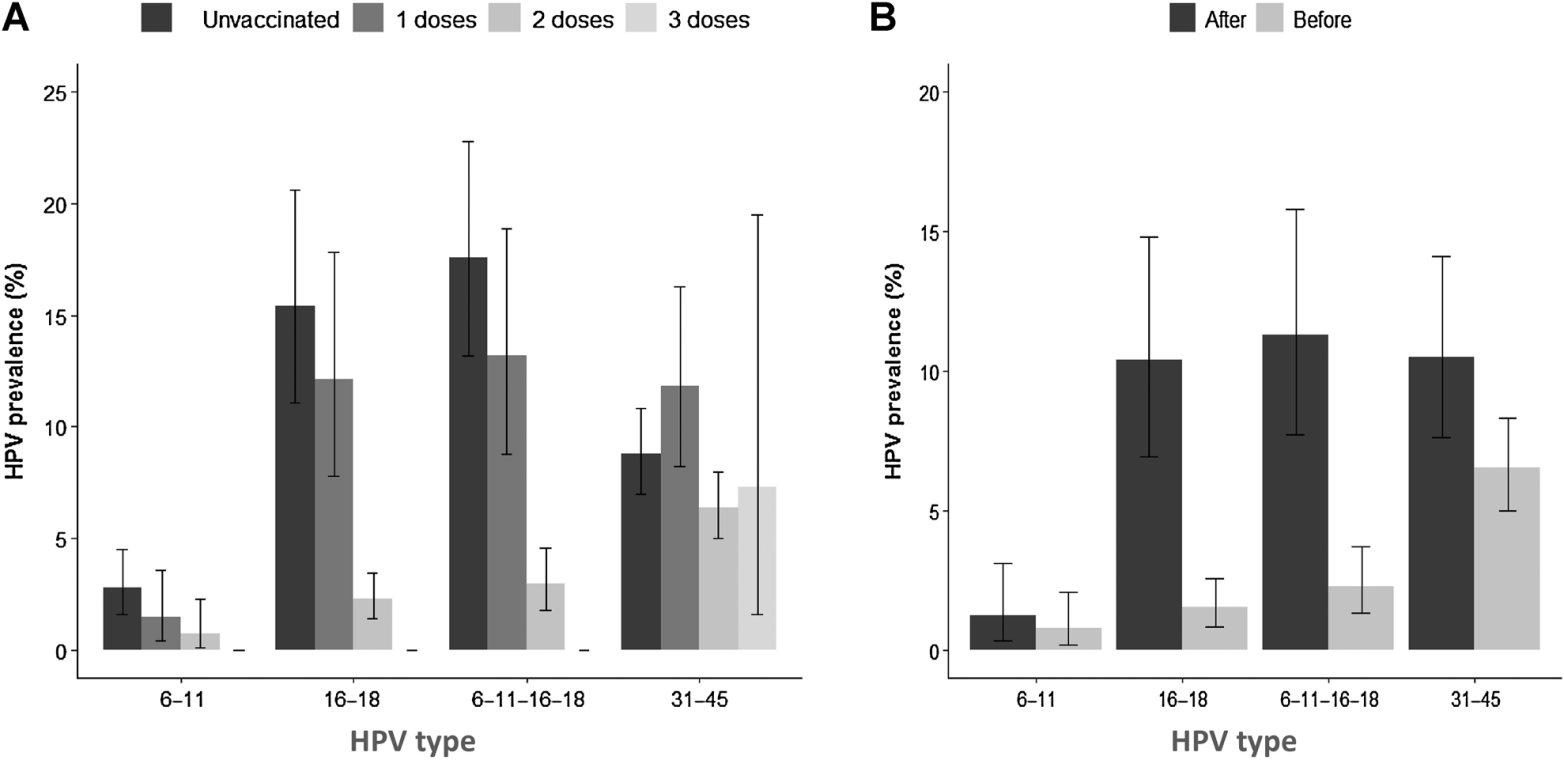
Prevalence of HPV vaccine types in the study population. Comparison between unvaccinated and vaccinates women ages 18 to 25 years according to doses (**A**) and to sexual debut (**B**).

As far as doses were concerned, after balancing by using the propensity score, complete protection to HPV vaccine types was observed with three doses (*P* < 0.00). Prevalence of HPV vaccine types with two doses decreased by 83.06% in vaccinated women, from 17.6% to 2.98% (*P* < 0.001). Similar changes in HPV prevalence rates were observed for HPV16–18 (15.4%–2.27%, *P* < 0.00), with a reduction of 85.5%. In contrast, with one dose, the observed reduction was only 25% for HPV vaccine types (17.6%–13.2%), and 21.4% for HPV 16/18 (15.4%–12.1%; Fig. 3A). In the same way, a statistically significantly reduced prevalence for HPV vaccine types in vaccinated women before sexual debut was observed, as compared with vaccinated women after sexual debut (2.29% vs. 11.3%, respectively). Similarly, regarding HPV16/18, there was a lower prevalence when women were vaccinated before sexual debut (1.52% vs. 10.4%, respectively; Fig. 3B). These differences were maintained after adjustment (*P* < 0.001).

### HPV vaccine effectiveness

**Figure 4.**
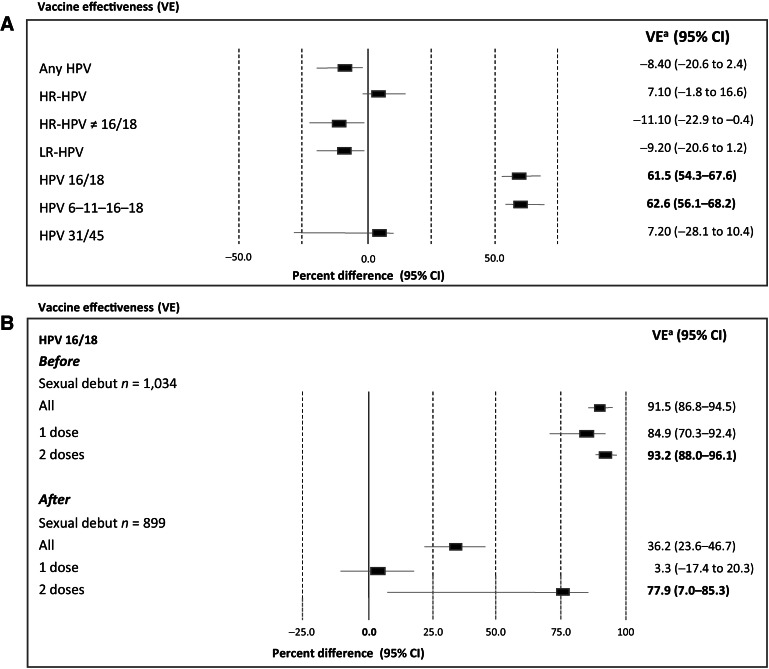
Vaccine effectiveness (VE) in vaccinated women aged 18–25 years: to different groups of HPV types (**A**) and to HPV16/18 according to sexual debut and number of doses (**B**). VE^a^Adjusted by using a propensity score analysis.

Figure 4 shows the vaccine effectiveness against HPV among young women after adjustment. The adjusted effectiveness against HPV16/18 type infections was 61.5, 95% CI, 54.3–67.6, while against all HPV types it was 62.6% for all HPV vaccines (62.5; 95% CI, 56.1–68.2), *P* < 0.001. No vaccine effectiveness against HR-HPV was observed: 7.1, 95% CI, 1.8–16.6), *P* > 0.05, neither against HR-HPV≠16/18: −11.10, 95% CI, −22.9 to −0.4, *P* > 0.05. Similarly, no vaccine effectiveness against HPV31 and 45 was observed: 7.2%, 95% CI, −28.1 to 10.4, *P* > 0.05 (Fig. 4A).

When vaccine effectiveness against HPV16–18 was assessed according to sexual debut and number of vaccine doses, a higher vaccine effectiveness was observed in women vaccinated before sexual debut (91.5%; 95% CI, 86.8–94.5), compared with those after sexual debut (36.2%; 95% CI, 23.6–46.7), being the highest after two doses (93.2%; 95% CI, 88.0–96.1, *P* < 0.001; Fig. 4B).

### HPV prevalence by pap smear result

Compared with unvaccinated women, the percentage of normal cytology was significantly higher in vaccinated women (90.5% vs. 93.7%), respectively, *P* = 0.002 (Supplementary Table S3). However, considering severity of cervical lesion as a clinical outcome, using ordinal logistic regression, no significant reduction was observed in the risk of cervical lesion (OR, 0.848; 95% CI, 0.683–1.054, *P* = 0.637).

**Figure 5.**
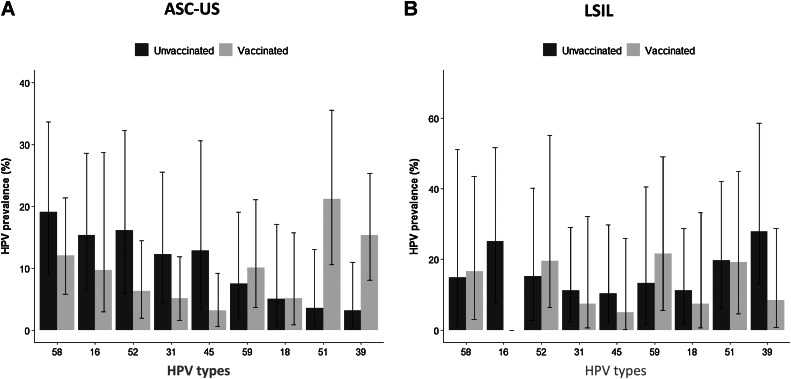
HPV prevalence by Pap smear result. Comparison of cytological abnormalities between unvaccinated and vaccinates women ages 18 to 25 years (**A**) prevalence of HPV types in ASC-US and (**B**) L-SIL. Adjusted by using a propensity score analysis.

Compared with unvaccinated women, the proportion of infections associated with HPV16 in women with normal cytology was lower in the vaccinated group (10.5% vs. 4.51%, respectively, *P* < 0.001). Similar results were observed for HPV18 (5.47% vs. 1.87%; *P* < 0.001; Supplementary Table S4). No differences in the prevalence of ASC-US among unvaccinated and vaccinated women were observed (4.13% vs. 3.78%, respectively). The proportion of ASC-US attributable to HPV16 was lower in vaccinated women (9.73%) compared with unvaccinated women (15.3%), although it was not significant (*P* = 0.68). No differences for HPV18 were observed between two groups. However, a significant reduction of ASC-US attributable to HPV45 in vaccinated compared with unvaccinated women was observed (3.15% vs. 12.9%, respectively, *P* = 0.05). In contrast, a significant increase of ASC-US associated with HPV39 and 51 was observed (*P* = 0.01 and *P* < 0.001, respectively; Fig. 5; Supplementary Table S4).

Finally, a minor prevalence of L-SIL in vaccinated compared with unvaccinated women was observed (0.83% vs. 1.68%, respectively), though it was not significant (0.49; 95% CI, 0.23–1.05), *P* = 0.06). However, no infection associated with HPV 16 in vaccinated women (0.0% vs. 28.5%, *P* = 0.01) was observed. Similarly, a lower proportion of L-SIL attributable to HPV18, 31, 45, and 39 was observed, but it was nonsignificant (Fig. 5; Supplementary Table S4). Interestingly, no ASC-US and L-SIL cases attributable to HPV16/18 were observed in women vaccinated with at least two doses before their sexual debut. Although an increase of L-SIL associated with HPV 59 was observed, it was not significant.

## Discussion

Monitoring HPV vaccines post-licensure plays an important role in assessing the progress of immunization programs, demonstrating the impact of vaccines on the population, and providing data for policy needs (16). This is the largest study conducted in sexually active women ages 18 to 25 years in Colombia five years after HPV vaccination program to assess the prevalence of vaccine HPV type infection. Consistent with data from clinical trials and monitoring programs, we found a reduced prevalence of all vaccine HPV type infections in vaccinated as compared with unvaccinated women, thus proving that the national HPV immunization program is successfully preventing HPV vaccine-type infections in sexually active young women in Colombia.

In line with other surveillance studies, which have reported an important reduction in prevalence for HPV6, 11, 16, and 18 in other populations, our study showed a substantial reduction in the prevalence of HPV vaccine types during the first 5 years after vaccine introduction in sexually active young women, providing evidence of direct protection by the quadrivalent vaccine, and suggesting high vaccine effectiveness in a real-world setting. In similar studies, a reduction of HPV16/18 from 19.1% to 6.5% among 16- to 18-year-old women (66% reduction) was reported in Great Britain (17). In Scotland, a 54% reduction in vaccine types has been shown in individuals ages 20 years (18), while in Sweden, surveillance among women attending for chlamydia screening found a reduction of 42% in HPV16 and 46% in HPV18 among females ages 13 to 22 years (19). Besides, an important fall in the prevalence of HPV vaccine types among women ages 18 to 24 years has been reported by Tabrizi and colleagues, where the prevalence of HPV16 decreased from 21.3% before vaccination to 4.9% some years after vaccination, whereas the prevalence of HPV18 decreased from 8.4% to 2.2% (20). Similarly, in the United States there was a significant decline in quadrivalent vaccine-type HPV among 13- to 26-year-old women, with a high degree of vaccine effectiveness when vaccinated women were compared with unvaccinated women (90.6% in wave 3 vs. wave 1, and 80.1% in wave 4 vs. wave 1; refs. 21, 22). In our study, we monitored 36 different HPV types, but only the HPV vaccine types showed a significant reduction, with vaccine effectiveness of 61.4% against HPV16/18 types. The differences in vaccine effectiveness may be associated with differences in vaccination coverage. In a mathematical modeling approach, it has been predicted that with 80% vaccination coverage, the vaccine effectiveness at the population level will result in a 78% reduction for HPV16 and 96% for HPV18; however, other factors like sexual mixing patterns, which markedly differ among different populations, could explain these differences (19, 23). In our study, the women included in the surveillance were vaccinated as part of a chat-up campaign, and although the vaccine had good coverage at the time, it is almost certain that some of those vaccinated may have had an existing HPV infection. Thus, the effectiveness assessed in the group of vaccinated women before sexual debut was higher (91.5%) as compared with 36.2% after sexual debut.

In our study, even though complete protection against HPV16/18 infections was observed in young women who received three doses of the HPV vaccine, we found that women who received two doses of vaccine had higher effectiveness (93.2) against HPV vaccine-type infection as compared with those who had one dose (84.9). These findings confirm the high effectiveness suggested by other studies, meaning that at least two doses are needed to achieve a high level of immunity across time and that it is still highly effective when given at older ages. Nevertheless, it has been found that young girls may have a better immunologic response to the HPV vaccine (2, 24–26). Thus, the reductions in the HPV vaccine types (HPV16/18) observed here reassure the benefits of HPV vaccination. Likewise, a decline in the prevalence of HPV6 and 11 in the age groups with high vaccination coverage has been reported for LR-HPV vaccine type (19). In agreement with these reports, a decline of HPV6 and 11 infections was found in our study. Although the prevalence of genital warts was not evaluated in this study, it is important for future studies to determine whether or not a decrease in genital warts is associated with these types.

Compared with the bivalent vaccine, the quadrivalent vaccine shows a more limited cross-protection, but a significant efficacy has been noted, mostly against HPV31 (27). In this study, no significant decrease of HPV31 and HPV45 infection was observed. Similar results were reported in a study conducted in Southern Sweden, in which a tendency toward decrease of HPV31 in the younger women group was observed, but it was not significant (19). Likewise, a significant reduction in ASCUS associated with HPV45 and a trend in reducing L-SIL associated with these two types were observed in our study. Hence, the results confirm the limited cross-protection associated with the quadrivalent vaccine. It is possible that the level of antibodies generated is not enough to induce a cross-protection. We also assessed the prevalence of the 5 additional HR types of the 9-valent vaccine, but unlike those reported by Heard, no differences in prevalence among vaccinated and unvaccinated women were observed. This difference is explained by cross-reactive to HPV31, 33, and 45 types found in Heard's study (28). These results suggest the need to include in future vaccination programs, the 9-valent or the bivalent HPV vaccine, which has shown substantial cross-protection against HPV31/33/45, and to a lesser extent against HPV35 and HPV58 up to 11 years postvaccination. These vaccines are an effective option in the prevention of preneoplastic lesions and cervical cancer. It has been shown to provide excellent protection against infection with these genotypes (29, 30).

Following a reduction in HPV vaccine-targeted genotypes, an increase in infection or disease due to other HPV types, not prevented by the vaccine, may occur because of reduced competition during natural infection (31). However, type replacement after vaccination is thought to be unlikely (11). Similar to other studies, no statistically significant differences were observed in the prevalence of other nonvaccine HR-HPV type infections between two groups, suggesting no evidence of type replacement. In contrast, a slight increase in some nonvaccine LR-HPV type, including HPV42, 62, 67, and 84, was observed, which suggests that other changes in population characteristics (sexual behavior) would increase their risk of acquiring a nonvaccine HPV type or it can be attributable to factors other than those measured, which may have been confounding factors and does not guarantee balance against unknown confounders.

In a previous study, Gray and colleagues reported a sporadic HPV39 and 51 occurrence, but no patterns suggestive of type replacement following vaccination with the bivalent HPV16/18 vaccine.

In the same way, in this study, we observed a significant increase in ASC-US associated with HPV39 and HPV51 infections; however, this increase was not observed in L-SIL. These results confirm that, although no conclusive signs of type replacement are observed, it is important to continue monitoring the incidence of HPV39 and HPV51 in future investigations (32). Lastly, although there has been no conclusive evidence to date of type replacement after HPV vaccine introduction, and given that they are still at high risk of cervical cancer with other HR-HPV types, these results highlight the importance of maintaining regular cervical cancer screening after vaccination in our population.

HPV16 and 18 types have been detected in about 34% of L-SIL in cytological abnormalities, reflecting that these low-grade lesions could indicate acute HPV infection (33, 34). In our study, we also examined the usefulness of HPV vaccine against cytological abnormalities, and we present early data suggesting the HPV vaccine impact on HPV16/18-related L-SIL in 18- to 25-year-old women in Colombia. Although a risk reduction of having cervical lesions was observed, this was not statistically significant; interestingly, no L-SILs associated with HPV16 were observed in the young women's group. These results are consistent with studies in Australia, where a population-based HPV vaccination program in schools significantly reduced cervical abnormalities, including low-grade cytology for vaccinated women within five years of implementation, with the greatest vaccine effectiveness observed for the youngest women from catch-up programs, which is a proxy for the onset of sexual activity (35).

The greatest impact of the HPV vaccine is expected to happen in low-income and middle-income countries in which there is no screening or only limited screening for cervical cancer and where the highest burden of cervical disease is generally observed. The impact of vaccination with current HPV vaccines in low resource settings, without any other preventive actions, is estimated to potentially reduce cancer risk by 40% to 50% with 70% vaccination coverage. The result of this study is an early indication that the national HPV program has been successfully preventing HPV16/18 infection in sexually active women, and is promising in terms of its implications for declining rates of CIN, and ultimately of ICC in the future. However, despite the successful uptake of the vaccine, the program was adversely affected by controversy and misinformation regarding its safety, and coverage has been in decline, specifically after the event of “El Carmen de Bolivar” (36). We hope that the data shown in this article may help reduce vaccine hesitancy in the Colombian population.

One of the strategies used to monitor the early impact of the HPV immunization program is the use of residual specimens from sexually active young women undergoing chlamydia screening (37). Yet, the young women screened for chlamydia tend to have a higher risk of chlamydia infection, and consequently higher risk of HPV infection than the general population, which likely increases power to detect changes but could limit the representativeness of the general population (37). In this study, we proposed a monitoring program based on a sentinel city-based surveillance study in young women under 25 years old attending higher education and technical centers. Moreover, the chosen city has a population cancer register indexed to the IARC, which will allow establishing a long-term impact of the vaccine against HPV on the incidence of CC. In our case, we have no evidence that participation was related to any factor associated with the HPV prevalence, other than the age group, which could limited the representativeness of the general population (37).

This study also has some limitations that should be interpreted with caution when addressed. First, this is a cross-sectional study carried out in two different periods, and even though strict comparability of samples between two periods is a general weakness of nonrandomized comparative studies, in this study, we collected the unvaccinated and vaccinated samples in the same geographic locations and in the same institutions. In an attempt to minimize differences in HPV risk-related characteristics between the two sample periods, we have adjusted all the available variables for comparing the two groups by using a propensity score analysis based on inverse probability of treatment weighting. We found that the decrease in HPV prevalence was maintained after adjusting the differences between the samples in demographic and other features. However, it is possible that differences in HPV prevalence may have been attributable to factors other than those measured in this study (11). On the other hand, one question that might arise from our finding is whether the large decrease in the prevalence of HPV vaccine genotypes, observed in samples of women recruited, could be extrapolated to the rest of the 18- to 25-year population of Colombia. Even though this is a preliminary study, and considering that further studies in other regions are necessary, these results should be interpreted with caution. Finally, this was a convenience sample, and it would be premature to draw conclusions about the impact of HPV vaccination on type replacement. Larger studies with more representative samples are needed to definitively address this issue.

In conclusion, this study provides a preliminary evaluation of the vaccine effectiveness against vaccine-targeted HR-HPV type infections following the implementation of the national HPV vaccination program in Colombia. In line with international data, five years after the vaccine was introduced in Colombia, we have shown a substantial and statistically significant decrease in the prevalence of vaccine HPV genotype infections. We did not find indications of cross-protection against HPV31/45. However, due to coverage decline in 2015, it is necessary to improve coverage rates among women and continuously monitor the vaccine impact, to evaluate if this decrease results in preventing preneoplastic lesions and invasive cervical cancer over time.

## Authors' Disclosures

No disclosures were reported.

## References

[1] Bruni L , Diaz M, Barrionuevo-Rosas L, Herrero R, Bray F, Bosch FX, . Global estimates of human papillomavirus vaccination coverage by region and income level: a pooled analysis. Lancet Glob Heal 2016;4:e453–63. 10.1016/S2214-109X(16)30099-7 27340003

[2] Garland SM , Lee Ly. Human papillomavirus vaccination: the population impact. F1000Research. 2017;6:1–11. 10.12688/f1000research.10691.1 PMC5473416 28663791

[3] Ministerio de Salud y Protección Social. Colombia. Vacunación contra el Virus Papiloma humano - VPH en Colombia, para la prevención del cáncer de cuello uterino y verrugas genitales. 2013; Available from: https://www.minsalud.gov.co/sites/rid/Lists/BibliotecaDigital/RIDE/IA/INCA/1-vacunacion-contra-virus-papilomahumano-verrugas-genitales.pdf.

[4] Vorsters A , Bosch FX, Bosch FX, Bonanni P, Franco EL, Baay M, . Prevention and control of HPV infection and HPV-related cancers in Colombia – a meeting report. BMC Proc 2020;14:1–13. 32577128 10.1186/s12919-020-00192-2 PMC7307134

[5] Colombia. Ministerio de Salud y Protección Social. HPV prevention and control board. Programa de vacunación contra el VPH en Colombia. 2018; Available from: https://www.uantwerpen.be/en/projects/hpv-prevention-and-control-board/meetings/hpv-colombia/.

[6] Simas C , Munoz N, Arregoces L, Larson HJ. HPV vaccine confidence and cases of mass psychogenic illness following immunization in Carmen de Bolivar, Colombia. Hum Vaccin Immunother 2019;15:163–6. 30118381 10.1080/21645515.2018.1511667 PMC6363158

[7] Martínez M , Estévez A, Quijada H, Walteros D, Tolosa N, Paredes A, . Brote de evento de etiología desconocida en el municipio de El Carmen de Bolívar, Bolívar, 2014. Inf Quinc epidemiológico Nac 2015;20:42–77.

[8] Wheeler CM , Hunt WC, Cuzick J, Langsfeld E, Pearse A, Montoya GD, . A population-based study of human papillomavirus genotype prevalence in the United States: baseline measures prior to mass human papillomavirus vaccination. Int J Cancer 2013;132:198–207. 22532127 10.1002/ijc.27608 PMC3852415

[9] Wong CA , Saraiya M, Hariri S, Eckert L, Howlett RI, Markowitz LE, . Approaches to monitoring biological outcomes for HPV vaccination: challenges of early adopter countries. Vaccine 2011;29:878–85. 20971113 10.1016/j.vaccine.2010.10.018

[10] Puerto D , Reyes V, Lozano C, Buitrago L, Garcia D, Murillo RH, . Detection and genotyping of HPV DNA in a group of unvaccinated young women from Colombia: Baseline measures prior to future monitoring program. Cancer Prev Res 2018;11:581–91. 10.1158/1940-6207.CAPR-17-0439 29991579

[11] Kahn JA , Widdice LE, Ding L, Huang B, Brown DR, Franco EL, . Substantial decline in vaccine-type human papillomavirus (HPV) among vaccinated young women during the first 8 years after HPV vaccine introduction in a community. Clin Infect Dis 2016;63:1281–7. 27655996 10.1093/cid/ciw533 PMC5091346

[12] Austin PC . An introduction to propensity score methods for reducing the effects of confounding in observational studies. Multivariate Behav Res 2011;46:399–424. 21818162 10.1080/00273171.2011.568786 PMC3144483

[13] Austin PC , Stuart EA. Moving towards best practice when using inverse probability of treatment weighting (IPTW) using the propensity score to estimate causal treatment effects in observational studies. Stat Med 2015;34:3661–79. 26238958 10.1002/sim.6607 PMC4626409

[14] Greifer N . Covariate balance tables and plots: a guide to the cobalt package [Internet]. 2019. Available from: https://cran.microsoft.com/snapshot/2020-04-20/web/packages/cobalt/vignettes/cobalt_A0_basic_use.html.

[15] Wilcosky TC , Chambless LE. A comparison of direct adjustment and regression adjustment of epidemiologic measures. J Chronic Dis 1985;38:849–56. 4044770 10.1016/0021-9681(85)90109-2

[16] Potter RC , Flagg EW, Datta SD, Saraiya M, Copeland G. Monitoring the impact of human papillomavirus vaccines on high-grade pre-invasive cervical lesions: designing a framework of linked immunization information system and cancer registry data in Michigan. Vaccine 2014;33:1400–5. 10.1016/j.vaccine.2014.12.063 PMC6921485 25573038

[17] Mesher D , Soldan K, Howell-Jones R, Panwar K, Manyenga P, Jit M, . Reduction in HPV 16/18 prevalence in sexually active young women following the introduction of HPV immunisation in England. Vaccine 2013;32:26–32. 24211166 10.1016/j.vaccine.2013.10.085 PMC3898718

[18] Kavanagh K , Pollock KGJ, Potts A, Love J, Cuschieri K, Cubie H, . Introduction and sustained high coverage of the HPV bivalent vaccine leads to a reduction in prevalence of HPV 16/18 and closely related HPV types. Br J Cancer 2014;110:2804–11. 24736582 10.1038/bjc.2014.198 PMC4037824

[19] Söderlund-Strand A , Uhnoo I, Dillner J. Change in population prevalences of human papillomavirus after initiation of vaccination: the high-throughput HPV monitoring study. Cancer Epidemiol Biomarkers Prev 2014;23:2757–64. 25380734 10.1158/1055-9965.EPI-14-0687

[20] Tabrizi SN , Brotherton JML, Kaldor JM, Skinner SR, Cummins E, Liu B, . Fall in human papillomavirus prevalence following a national vaccination program. J Infect Dis 2012;206:1645–51. 23087430 10.1093/infdis/jis590

[21] Ding L , Children C, Brown D. Human papillomavirus vaccine effectiveness and herd protection in young women. Pediatrics 2019;143:e20181902. 30670582 10.1542/peds.2018-1902 PMC6361347

[22] Kahn JA , Brown DR, Ding L, Widdice LE, Shew ML, Glynn S, . Vaccine-type human papillomavirus and evidence of herd protection after vaccine introduction. Pediatrics 2012;130:249–56. 10.1542/peds.2011-3587 PMC3408690 22778297

[23] Vänskä S , Auranen K, Leino T, Salo H, Nieminen P, Kilpi T, . Impact of vaccination on 14 high-risk HPV type infections: a mathematical modelling approach. PLoS One 2013;8:e72088. 24009669 10.1371/journal.pone.0072088 PMC3756967

[24] McClung NM , Gargano JW, Bennett NM, Niccolai LM, Abdullah N, Griffin MR, . Trends in human papillomavirus vaccine types 16 and 18 in cervical precancers, 2008–2014. Cancer Epidemiol Biomarkers Prev 2019;28:602–9. 30792242 10.1158/1055-9965.EPI-18-0885 PMC6526945

[25] Gee J , Naleway A, Shui I, Baggs J, Yin R, Li R, . Monitoring the safety of quadrivalent human papillomavirus vaccine: findings from the vaccine safety datalink. Vaccine 2011;29:8279–84. 21907257 10.1016/j.vaccine.2011.08.106

[26] Guo F , Hirth JM, Berenson AB. Comparison of HPV prevalence between HPV-vaccinated and non-vaccinated young adult women (20–26 years). Hum Vaccines Immunother 2015;11:2337–44. 10.1080/21645515.2015.1066948 PMC4635939 26376014

[27] Malagón T , Drolet M, Boily MC, Franco EL, Jit M, Brisson J, . Cross-protective efficacy of two human papillomavirus vaccines: a systematic review and meta-analysis. Lancet Infect Dis 2012;12:781–9. 22920953 10.1016/S1473-3099(12)70187-1

[28] Heard I , Tondeur L, Arowas L, Demazoin M, Falguières M, Du Chatelet IP. Effectiveness of human papillomavirus vaccination on prevalence of vaccine genotypes in young sexually active women in France. J Infect Dis 2017;215:757–63. 28011911 10.1093/infdis/jiw639

[29] Tsang SH , Sampson JN, Schussler J, Porras C, Wagner S, Boland J, . Durability of cross-protection by different schedules of the bivalent HPV vaccine: the CVT trial. J Natl Cancer Inst 2020;112:1030–7. 32091596 10.1093/jnci/djaa010 PMC7566371

[30] Serrano B , Alemany L, Tous S, Bruni L, Clifford GM, Weiss T, . Potential impact of a nine-valent vaccine in human papillomavirus related cervical disease. Infect Agent Cancer 2012;7:1–13. 23273245 10.1186/1750-9378-7-38 PMC3554470

[31] Schuchat A , Bell BP. Monitoring the impact of vaccines postlicensure: new challenges, new opportunities. Expert Rev Vaccines 2008;7:437–56. 18444891 10.1586/14760584.7.4.437

[32] Gray P , Palmroth J, Luostarinen T, Apter D, Dubin G, Garnett G, . Evaluation of HPV type-replacement in unvaccinated and vaccinated adolescent females—post-hoc analysis of a community-randomized clinical trial (II). Int J Cancer 2018;142:2491–500. 29377141 10.1002/ijc.31281

[33] Guan P , Howell-Jones R, Li N, Bruni L, De Sanjosé S, Franceschi S, . Human papillomavirus types in 115,789 HPV-positive women: a meta-analysis from cervical infection to cancer. Int J Cancer 2012;131:2349–59. 22323075 10.1002/ijc.27485

[34] Clifford GM , Rana RK, Franceschi S, Smith JS, Gough G, Pimenta JM. Human papillomavirus genotype distribution in low-grade cervical lesions: comparison by geographic region and with cervical cancer. Cancer Epidemiol Biomarkers Prev 2005;14:1157–64. 15894666 10.1158/1055-9965.EPI-04-0812

[35] Gertig DM , Brotherton JML, Budd AC, Drennan K, Chappell G, Saville AM. Impact of a population-based HPV vaccination program on cervical abnormalities: a data linkage study. BMC Med 2013;11:1–12. 24148310 10.1186/1741-7015-11-227 PMC4015688

[36] Cordoba-Sanchez V , Tovar-Aguirre OL, Franco S, Arias Ortiz NE, Louie K, Sanchez GI, . Perception about barriers and facilitators of the school-based HPV vaccine program of Manizales, Colombia: a qualitative study in school-enrolled girls and their parents. Prev Med Reports 2019;16:100977. 10.1016/j.pmedr.2019.100977 PMC6722392 31508297

[37] Mesher D , Panwar K, Thomas SL, Beddows S, Soldan K. Continuing reductions in HPV 16/18 in a population with high coverage of bivalent HPV vaccination in England: An ongoing cross-sectional study. BMJ Open 2016;6:1–8. 10.1136/bmjopen-2015-009915 PMC4762111 26868944

